# Supplementary material for: A unified moment tensor potential for silicon, oxygen, and silica
Source: NPJ Comput Mater. 2024 Sep 13;10(1):218. doi: 10.1038/s41524-024-01390-8 (PMC11399103; doi:10.1038/s41524-024-01390-8)
Supplement: Supplementary file 1 — Supplementary Information for: a unified moment tensor potential for silicon, oxygen, and silica [file 41524_2024_1390_MOESM1_ESM.pdf]

Supplementary Information for:

A unified moment tensor potential for silicon, oxygen, and silica

Karim Zongo, Hao Sun, Claudiane Ouellet-Plamondon, and Laurent Karim Béland

Database preparation

The ab initio reference data plays a crucial role as one of the main components in machine learning inter-atomic potential (MLIAP) methods. The success of an MLIAP in achieving near DFT-accuracy and transferability relies not only on the model itself but also on the quality and characteristics of the reference data used. The features of the reference data should encompass the quality of the quantum mechanical calculations, the diversification of atomic configurations, and the coverage of the configurational space. To construct a data-driven reactive force field, it is essential to consider various bonding and coordination scenarios, including charge transfer. This is made possible by examining polymorphs of the materials and conducting AIMD simulations at different temperatures and pressures. For instance, the coordination number changes from 4 in cubic diamond silicon to 6 in beta-tin and hexagonal silicon at higher pressure [1]. Some of the silicon polymorphs exhibit varying bond angles in their minimum energy state [2, 3]. Another example includes silica polymorphs, where silicon and oxygen are typically four-fold and two-fold coordinated, except in the case of seifertite and sisthovite structures, in which silicon is six-fold coordinated and oxygen is three-fold coordinated, respectively. In addition, during molecular dynamics simulations, over/under coordination is sometime inevitable. Thus, we have considered diverse configurations, including crystal structures, strained crystal structures, point defects, extended defects, and disordered structures, in which our materials of interest may chemically exist. Indeed, these configurations represent different states or phenomena of the materials under external conditions. Most of the phenomena, such as defect migration mechanisms, defect stability, and phase changes, were considered in the construction of our database. We followed the procedure described in [4] to generate our database. However, in this article, several additional techniques and methods were incorporated to complement the ones described in [4] to create a comprehensive database of Si/SiO_2/O. The construction of the database involved various types of calculations, including single point calculations, geometry optimizations, and ab initio molecular dynamics simulations (AIMD). The final step of each geometry optimization was considered and included in the database. For the AIMD simulations, we followed a two-step process. In the first step, we performed

AIMD simulations with low values of parameters, such as k-points and kinetic energy cutoff (ecutwfc). For instance, we set the kpoints to 1 1 1 and energy criterion for convergence of self-consistent field (SCF) iterations to 10^{-8} eV. In the second step, snapshots were taken at intervals of 100 time steps to minimize correlation. The selected configurations were then recalculated with higher values of k-points and kinetic energy cutoff (ecutwfc). All the calculations were performed using the Quantum Espresso package. The majority of numerical details can be located in the "Ab Initio Calculation Details" section of the main article. We consulted multiple databases, including the Materials Project [5], the American Mineralogist Crystal Structure Database [6], and the Automatic Flow for Materials Discovery (AFLOW) [7]. The majority of our crystal structures, which are labeled with atomic coordinates and lattice parameters, are sourced from experimental X-ray diffraction (XRD) or quantum mechanical calculations available in these databases. Subsequently, we utilized these inputs for geometry relaxation, employing thoroughly converged density functional theory (DFT) parameters. It is important to highlight that the development of the comprehensive database and molecular dynamic simulations was made possible through the use of various modeling packages. Among them were Atomsk [8], Vesta [9], Ovito [10], and Xcrysden [11].

Silicon

We considered nearly 13 polymorphs of silicon, along with the liquid and amorphous states. While the majority of the process for generating the silicon database can be found in [4], additional calculations were conducted in this study to enhance the completeness of the database. Ab initio molecular dynamics simulations were conducted using a supercell of 63 atoms containing a vacancy at five different temperatures: 400, 800, 1600, 2600, and 3600 K. In addition to the liquid configurations generated in [4], we utilized a MTP-trained potential to melt the silicon at temperatures of 2000 K and 3000 K. The resulting melted configurations were then used as inputs for the subsequent AIMD equilibration. Although in [4], AIMD liquid configurations were equilibrated near these temperatures, in this study we used different starting inputs.The aim is to achieve a high degree of diversification in the atomic environments. In contrast to the random displacement configurations used as input for the AIMD melting process in [4], we utilized the MTP-melts as the initial input for the AIMD equilibration in this work. Two models were utilized in this study: one with 64 atoms and another with 216 atoms. These models were gradually heated over a period of 30 ps and subsequently equilibrated for 20 ps using the Stillinger-Weber potential. The resulting liquid structure at each temperature was then used as input for the ab initio molecular dynamics (AIMD) simulation. The AIMD simulations employed a time-step of 1 fs for the 64-atom model and 2 fs for the 216-atom model. For example, the result of the MTP potential-based melting at 2000 K was used as the input for the subsequent AIMD equilibration at 2000 K. Compressed liquid was also incorporated in the database by filling a cubic box with 64 atoms in order to achieve a density of 3.20 g/cm^3. We also included a small model for AIMD liquid silicon using a unit cell of the diamond structure containing 8 atoms. This model was equilibrated at 2500 K and 3500 K. Additional models were included to simulate amorphous silicon. A cubic box was randomly filled with 120 atoms, aiming for a density equal to the experimental density of amorphous silicon (2.28 g/cm^3). The configuration was then briefly equilibrated using the Stillinger-Webber [12] potential before the AIMD run.

Silica

The silica database was constructed by including all its polymorphs and deformations, following the Voigt notation, similar to the approach used for silicon. In addition, disordered structures were included in the SiO_2 database through a two-step process. First, pre-relaxation was performed using the Beest Kramer van Santen (BKS)[13] potential, followed by full equilibration using DFT calculations. The density was

adjusted to 2.20 g/cm^3 in each process to match the experimental density. This was achieved by utilizing various cubic box models containing 3, 6, 9, 12, 27, 36, 48, 54, 72, 96, 114, and 192 atoms. The silicon and oxygen atoms were randomly distributed within these boxes according to the stoichiometry. The preliminary relaxation was conducted using the BKS potential at various temperatures ranging from 300 K to 6000 K. Each configuration was gradually heated to the target temperature over a period of 80 ps and subsequently equilibrated for 30 ps using a time step of 1 fs. Each result obtained from the preliminary relaxation was used as input for AIMD equilibration at the corresponding BKS relaxation temperature. As an example, the result of the BKS pre-equilibration at 3000 K was utilized as the input for the subsequent AIMD equilibration at the same temperature. The time step for all AIMD equilibrations was set to 2 fs. The maximum number of time steps recorded was 35 ps. Additionally, random displacements were introduced within the selected configurations at temperatures ranging from 300 K to 1000 K.

Oxygen

The oxygen database, which accounts for 2% of the full database, was constructed by considering only the gaseous state. This choice was made because PBE-based DFT modeling has limitations in accurately describing solid oxygen, where both van der Waals and covalent interactions coexist. Thus, for gaseous phases such as dioxygen, ozone, and others, we first optimized the bond length and bond angle. Then, we varied the bond length of the diatomic molecule and computed the corresponding properties. In the case of bent molecules like ozone, we varied the bond angle and performed two types of calculations. In the first set of calculations, we kept the bond length fixed while varying the bond angle. In the second set of calculations, we optimized the bond length at each angle. Additionally, we added oxygen configurations from chemical reactions using the nudge elastic band method (NEB) [14]. Furthermore, we selected other types of structures, such as oxygen clusters and silicon dimers, from the silica melt at higher temperatures using the MTP potential.
For a comprehensive breakdown of the database composition, please consult Tab S8 and [4].

Active learning

In addition to the database generation methods described above, we performed additional techniques to enhance and facilitate the training of the unified potential for the Si-SiO_2 and O system. First to improve our potential and diversity of our database, we added interfacial configurations of Si-SiO_2. We built few models of Si-SiO_2 interface and conducted single point calculations of these models. We also equilibrate these configurations shortly using AIMD simulation at 300 K, 500 K and 800 K and selected fews configurations from the short trajectories and compute their energies, forces and stress with DFT. We then merged these configurations to ones from single point calculations of the unrelaxed models and trained with low level MTP potential (level 08). The resulting potential were used to conduct active learning. The active learning approach implemented in the MTP framework uses the D-optimality criterion [15, 16]. This criterion allows for the detection of configurations on which the MTP potential extrapolates during MD simulations. We run MD trajectories at 300 K and 800 K for 100 ps and extrapolative configurations were gathered to complement our database need. In principle, during a 100 ps simulation, the MTP potential is retrained on the fly. However, in this case, we are not interested in the retrained potential; instead, we focus on the extrapolative configurations. As our primary database contain a lot of large models mainly for vacancy, divacancy and interstitial, we did an active learning on these defects using unit cell of 7, 6, 9 atoms built from the diamond silicion unit cell that contain 8 atoms. The same procedure elaborated for interfaces was used here. First, starting with an 8-atom unit cell, we either removed 1 or 2 atoms or added 1 atom. We then conducted SFC calculations and relaxed the resulting configurations. Thus, for each defect, we

obtained 2 configurations, which we used to fit our MTP 08 potential and begin the active learning process at 300 K. For interstitials, separate active learning was conducted for each of the interstitial positions, including bond-breaking interstitial, hexagonal interstitial, and tetragonal interstitial.

Training mode

One of the central challenges in developing a machine learning interatomic potential is the proper optimization of the cost function, which encompasses a multitude of parameters. The number of these parameters typically varies based on the data's characteristics, encompassing configuration types, their quantity, and the distribution of configuration sizes. Given the non-uniform distribution of sizes within the dataset, we employ two distinct training modes: the vibration mode and the structure mode. In the initial phase, we train the clean potential using the default vibration mode, as implemented in the MTP code. In this mode, an equal significance is given to every force vector, irrespective of the size distribution among configurations. This pertains to the simulation of thermal properties within the context of a molecular dynamic simulation. In the second step, we train the potential obtained from the first mode using structure modes. All the configurations have equal weight regardless of the size distribution. This seems to resemble a normalization. We have found the two-step training approach to be highly valuable mainly for calculating cohesive energy. From Fig. S8, it is evident that the energy error on isolated atoms is never eliminated when using the vibration mode, whereas the error becomes zero when the structure mode is employed.

Amorphous silicon

A 1000-atom cubic simulation box was used. For all the potentials, the configuration was heated to 3000 K and equilibrated for 30 ps. The configuration was then cooled to 1800 K at a cooling rate of 10 K/ps and equilibrated at 1800 K for 30 ps. Following this, the configuration was cooled to 1300 K at a cooling rate of 8.5 K/ps and equilibrated at 1300 K for another 50 ps. Finally, the configuration was cooled from 1300 K to 500 K at a rate of 5 K/ps and equilibrated at 500 K using the NPT ensemble for 120 ps. Structural data were gathered for the last 20 ps.

Amorphous silica

The configuration is heated from 300 K to 4200 K at a heating rate of 65 K/ps. We then scale the velocity to exactly 4200 K and equilibrate for 10 ps. After that, the velocity is scaled again to 3500 K, and the system is equilibrated for 35 ps at 3500 K. From 3500 K, the configuration is cooled to 2000 K at a cooling rate of 25 K/ps and equilibrated at 2000 K for 50 ps. Finally, the configuration is cooled to 300 K at a cooling rate of 10 K/ps and equilibrated with the NPT ensemble for 120 ps at 300 K. The structural properties were measured during the last 20 ps. The characteristics of the obtained structure match those of the experimental data, as we reported both in the main text and here. The experimental elastic constant of amorphous silica [17] are C_{11} = 77.93 GPa, C_{12} = 15.63 GPa and C_{44} = 31.15 GPa. Our MTP predicted elastic constant are C_{11} = 76.08 GPa, C_{12} = 18.90 GPa and C_{44} = 38.36 GPa. To ensure effective utilization of most semi-empirical potentials and achieve complete melting of the crystalline β-cristobalite, a specific heating and cooling protocol was implemented. The procedure for all semi-empirical models is as follows: The configuration is initially heated from 300 K to 5000 K over 60 ps. It is then held at 5000 K for 100 ps before being cooled to 3000 K at a rate of 20 K/ps. Once at 3000 K, the configuration is equilibrated for 50 ps, after which it is cooled to 2000 K at the same rate of 20 K/ps. Following this, another equilibration period of 50 ps at 2000 K is conducted as in MTP case. Finally, the configuration is cooled to 300 K at a

rate of 10 K/ps. It is then equilibrated in the NPT ensemble at 300 K for 120 ps, during which structural data is collected for the last 20 ps.

Tab. S1: Examples of fitting parameters for levels 26 and 28 of MTP potentials, consisting of 1657 and 2638 parameters respectively, are provided for a two-component system.
The table highlights both the hyperparameters of the MTP model and the free parameters optimized during the training phase. The hyperparameters, such as radial functions count, radial basis size, interaction cutoff radius, and weight, are manually set before the training process commences. Selecting the right hyperparameters is crucial for achieving the desired performance of the model. In addition to the primary hyperparameters, careful attention to the BFGS iteration number becomes pivotal. This parameter precisely dictates the frequency with which the model undergoes updates through the Broyden–Fletcher–Goldfarb–Shanno (BFGS) iterative algorithm. The impact of this parameter on the convergence and efficiency of the optimization process is particularly pronounced when considering the number of model parameters. Choosing appropriate values for this parameter is paramount, as excessively low values (less training time) may result in underfitting, while overly high values (more training time) can lead to overfitting. Striking the right balance is key, as there exists an optimal number for this parameter that ensures the model converges effectively without sacrificing its ability to generalize.

Parameters	Values (level 26)	Values (level 28)
radial functions count f_μ	6 (μ = 6)	6 (μ = 6)
radial basis size $Q^{(\alpha)}$	8 (α = 8)	8 (α = 8)
free parameters	1657	2638
Cutoff radius (Å)	5.7	5.7
Energy weight	1	1
Force weight	0.01	0.01
Stress weight	0.001	0.001
BFGS iteration number	3000	2000
Structures weighting mode	vibrations	structures
Parameters initialization mode	random	random

Tab. S2: The computational expenses.
The expense is assessed through the execution of a molecular dynamics (MD) simulation, employing a system comprising 64 atoms for silicon and 96 atoms for silica. The simulation with MTP and semi-empirical potentials is specifically configured to use a single CPU core, simplifying the comparison process. The cost is given in seconds per MD step

Silicon		Silica			
method	times	method	times	method	times
DFT	118	DFT	16.51	SHK1	0.0016
MTP26	0.062	MTP26	0.16	SHK2	0.0016
MTP28	0.138	MTP28	0.25	TS	0.0003
SW	0.0002	BKS	0.0015	VA	0.0005

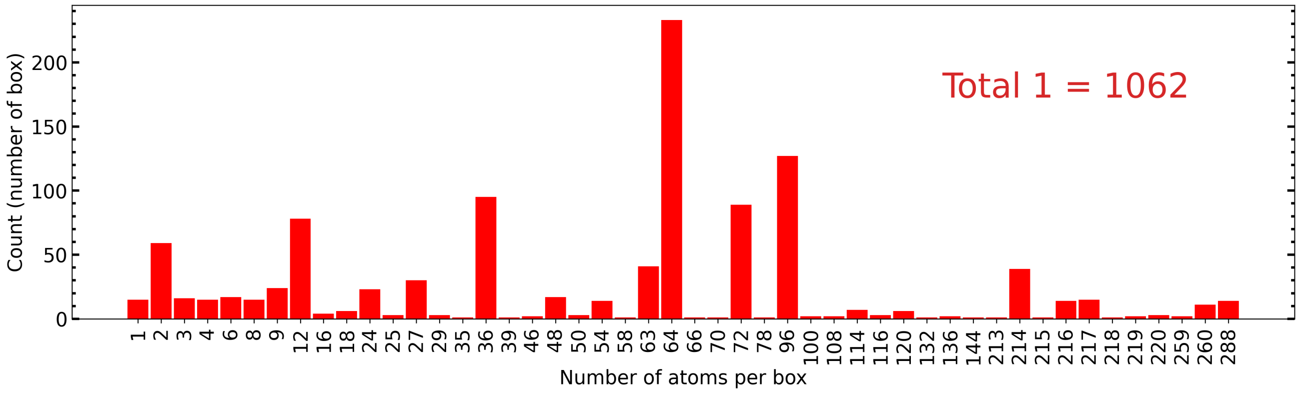

(a)

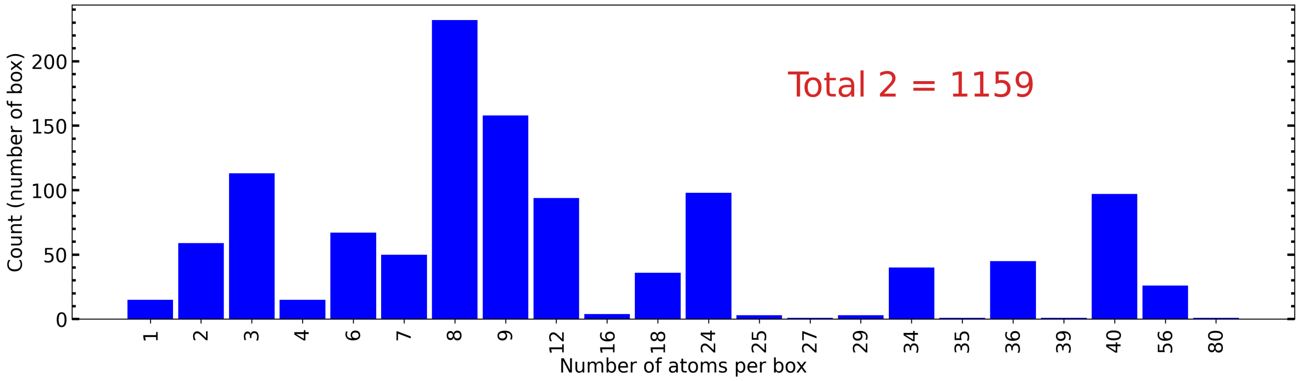

(b)

Fig. S1: Distribution of configuration sizes in the unified training sets ($T_{unified}$): (a) for preliminary work, testing, and training; (b) for final implementation. Total 2 = 0.52 x Tolal 1 + new DFT calculations .

The first training set Fig. S1 (a) corresponds to configurations ranging in size from 1 to 288 atoms, without any interfaces. For this set, we implemented a potential of level 26 (pot26.mtp) from the GitLab repository. This set was part of optimizing the database and testing the training mode. Given that we used different types of configurations, the distribution of the configuration size dictates the settings for training, including the training mode and the number of parameters. This allows us to choose a normalization approach, ensuring that all configurations have the same relative importance. Ensuring a diverse distribution of sizes and various configuration types in the dataset is instrumental in enabling the model to acquire knowledge of different atomic environments of the configurational space. This diversity is essential for enhancing the robustness of the interaction potential. However, this introduces a layer of complexity to the decision-making process, particularly when determining the necessary number of parameters for training the model. Given the utilization of various configuration types and the highly distributed configuration sizes, scaling the number of parameters in the model becomes a non-trivial task, as it cannot be directly determined based on the size of the training set. To enhance our work and predict properties more accurately, we used a second training set (Fig. S1 (b)) and trained with level 28 (pot28.mtp). Because the configurations are relatively small, we trained directly using the structure mode. The results in the main article correspond to this set, which has a total of 1149 configurations. The first set, with a total of 1062 configurations, was used for preliminary work and testing. Total 2 is made up of 52% of the configurations from Total 1, plus new DFT calculations (active learning). Please refer to Tab. S7 for the composition of Total 1 and to Tab. V from the main article for the composition of Total 2.

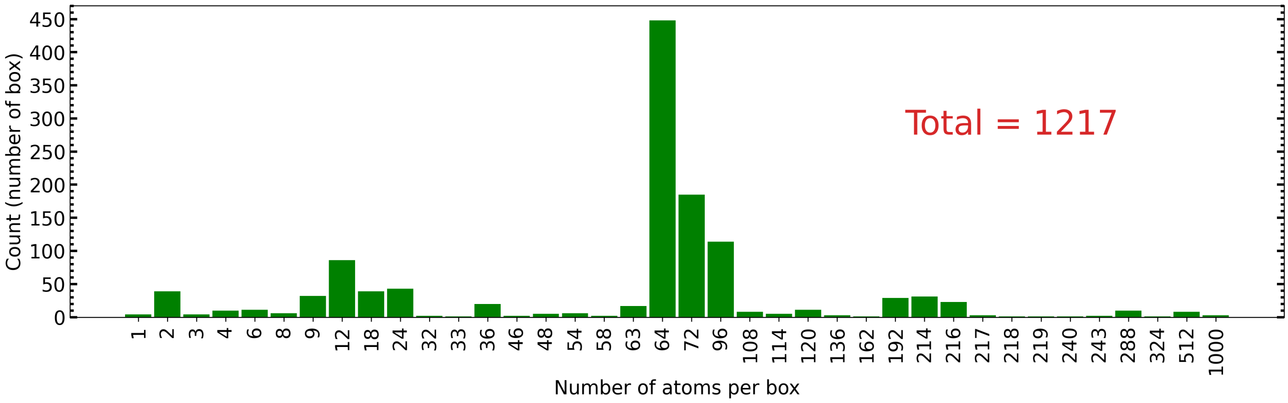

Fig. S2: Distribution of configuration sizes in the validation set V_1 picked from the curated data such that $V_1 \cap T_{unified} = \emptyset$.
Analyzing the distribution of size and configuration types in the validation set enables us to proficiently assess potential issues of underfitting or overfitting during the training task. The distribution of configuration sizes in this set is more varied compared to the unified training set.

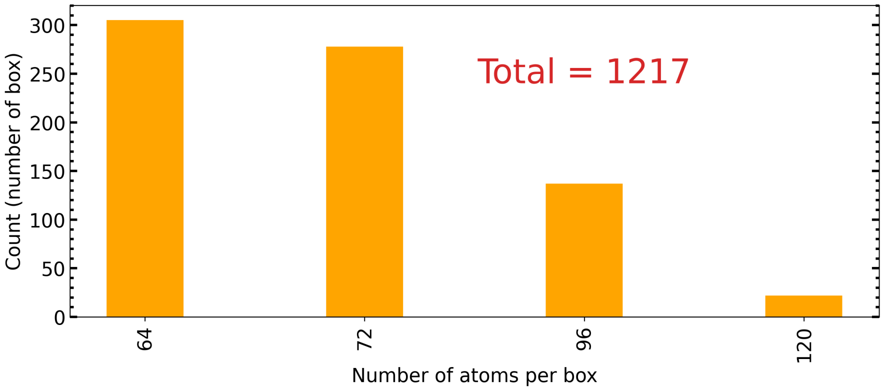

Fig. S3: Distribution of configuration sizes in the validation set V_2 selected from AIMD cooling trajectories.
In addition to the initial validation set mentioned earlier, this analysis allows us to assess the transferability capability of the MTP potential. Notably, configurations from this set were excluded from the training phase, no configurations from the AIMD cooling trajectory were incorporated into the training set.

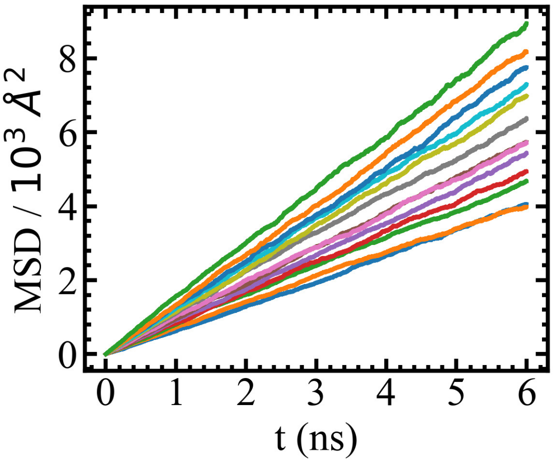

Fig. S4: Mean square displacement.
The time dependence of mean square displacement in silicon crystal with a vacancy across a temperature range from 1000 K to 1650 K. Based on this dynamical correlation function, we calculate the diffusion coefficient via the Einstein relation and determine the activation energy for diffusion. TThis is crucial as it allows for a comparison with NEB calculations. The MTP results, along with those from SW, are detailed in the main paper.

Tab. S3: Illustrations of training errors (RMSE) on defect subsets
The symbol E represents the energy error per atom in meV/atom, and F represents the force error in meV/Å. The table displays the ultimate training results following multiple iterations of our data cleaning methods, namely, the train-remove-train process. The last column (D1) represents the outcome of training on the combined, cleaned defects subsets. The level of the potential are also shown.

	Vacancies / divacancies Total = **51** min 63 max 215 mean 214		Interstials Total = **34** min 217 max 260 mean 217		Stacking fault Total = **40** min 36 max 36 mean 36		Others Total = **21** min 8 max 288 mean 288		D_1 Total = **146** min 8 max 288 mean 156	
Level	E	F	E	F	E	F	E	F	E	F
08	-	-	-	-	-	-	4.2	160.0	-	-
10	4.7	66.0	5.4	47.9	5.2	69.2	1.5	37.1	-	-
12	3.2	60.0	4.4	47.0	4.5	62.4	-	-	9.0	121.0
14	-	-	-	-	-	-	-	-	9.1	100.0

Tab. S4: Illustrations of training errors (RMSE) on cohesive energy and elastic constant subsets
These subsets were assembled from deformed configurations, incorporating some unstrained minimum energy states. Their primary purpose is to facilitate the calculation of elastic constants. Once more, we solely present the training results from the last iteration of the cleaning process.

	Elastic constant Cohesive energy Si EC_1 Total = **111** min 1 max 64 mean 10		Elastic constant Cohesive energy SiO_2 EC_2 Total = **161** min 3 max 36 mean 12		Others OC_1 Total = **45** min 2 max 132 mean 35		EC_1 + EC_2 Total = **272** min 1 max 36 mean 12		T1 = EC_1 + EC_2 + OC_1 Total = **317** min 1 max 132 mean 16	
Level	E	F	E	F	E	F	E	F	E	F
10	8.7	34.2	5.7	64.2	-	-	-	-		
12	5.4	21.7	4.3	53.5	8.2	202.8	10.4	64.4	17.5	215.6
14	-	-	-	-	5.3	188.5	8.8	45.1	11.3	168.1
16	-	-	-	-	-	-	-	-	9.6	125.1

Tab. S5: Illustrations of training errors (RMSE) on disordered structures subsets.
These are the results of the final iteration of the cleaning process applied to disordered silicon and silica. We cleaned disordered silicon and silica separately. The configurations in these subsets were obtained from AIMD simulations at various temperatures, as explained above. For silicon structures, we applied a selection (select-add command), while for silica, we initially utilized a train-remove-train process to exclude off-equilibrium configurations resulting from high-temperature AIMD simulations, followed by the application of the selection

	AIMD Si AL_1 Total = **272** min 63 max 216 mean 64		AIMD SiO_2 AL_2 Total = **325** min 27 max 114 mean 96		T2 = AL_1 + AL_2 Total = **597** min 27 max 216 mean 72	
Level	E	F	E	F	E	F
16	5.3	136.0	4.0	178.6	7.2	217.8
18	5.1	133.2	4.4	172.1	5.6	189.3

Tab. S6: The first unification process that led to training set 1 (see Total 1 in Fig. S1 (a) for the distribution of atoms and Tab. S7 for the composition) and validation sets

Training error measured on the unified training set 1 and validation error, both expressed as (RMSE), with E representing energy error per atom in meV/atom, F representing force error in meV/Å, and S representing stress error in GPa. Additionally, we performed joint training on defect structures, elastic constants, and molecules, denoted as set T_3. Subsequently, isolated atoms were introduced to observe their impact on the optimization process. Finally, we augmented the training set by incorporating disordered structures of silicon and silica, resulting in the formation of the first unified training set (Total 1) . We utilized the validation set to assess the underfitting/overfitting and generalization capabilities of the unified potentials. The notations R and S represent random mode and structure mode, respectively.

	$T_3 = D_1 + T_1$ Total = **463** min 1 max 288 mean 68		$T_4 = T_3 + T_{isolated}$ Total = **465** min 1 max 288 mean 68		$Tf = T_4 + T_2$ Total 1 = **1062** min 1 max 288 mean 67			V_1 Total = **1217** min 1 max 1000 mean 75			V_2 Total = **742** min 1 max 120 mean 74		
Level	E	F	E	F	E	F	S	E	F	S	E	F	S
18 R	11.7	85.7	16.2	100.0	36.7	220.1	2.5	22.8	278.754	1.0	25.3	199.4	0.6
20 R	9.1	79.0	9.1	71.9	17.5	174.9	1.5	17.3	220.2	0.9	19.3	156.4	0.3
22 R	7.7	63.2	7.3	66.6	14.2	153.6	0.9	17.1	216.1	0.6	24.6	146.3	0.3
24 R	-	-	-	-	10.1	135.2	0.7	11.9	187.4	0.5	20.3	135.7	0.2
26 R					9.7	123.1	0.6	11.8	202.8	0.5	18.8	123.8	0.2
18 S					13.9	226.2	0.9	16.1	283.1	0.7	16.2	204.2	0.4
20 S					9.9	178.7	0.7	11.5	224.7	0.6	16.2	157.4	0.3
22 S					8.3	157.7	0.5	10.8	213.5	0.5	17.5	149.3	0.3
24 S					6.3	137.3	0.4	8.2	191.9	0.4	17.0	139.4	0.2
26 S	-	-	-	-	5.4	126.0	0.3	8.3	208.0	0.4	15.2	126.6	0.2

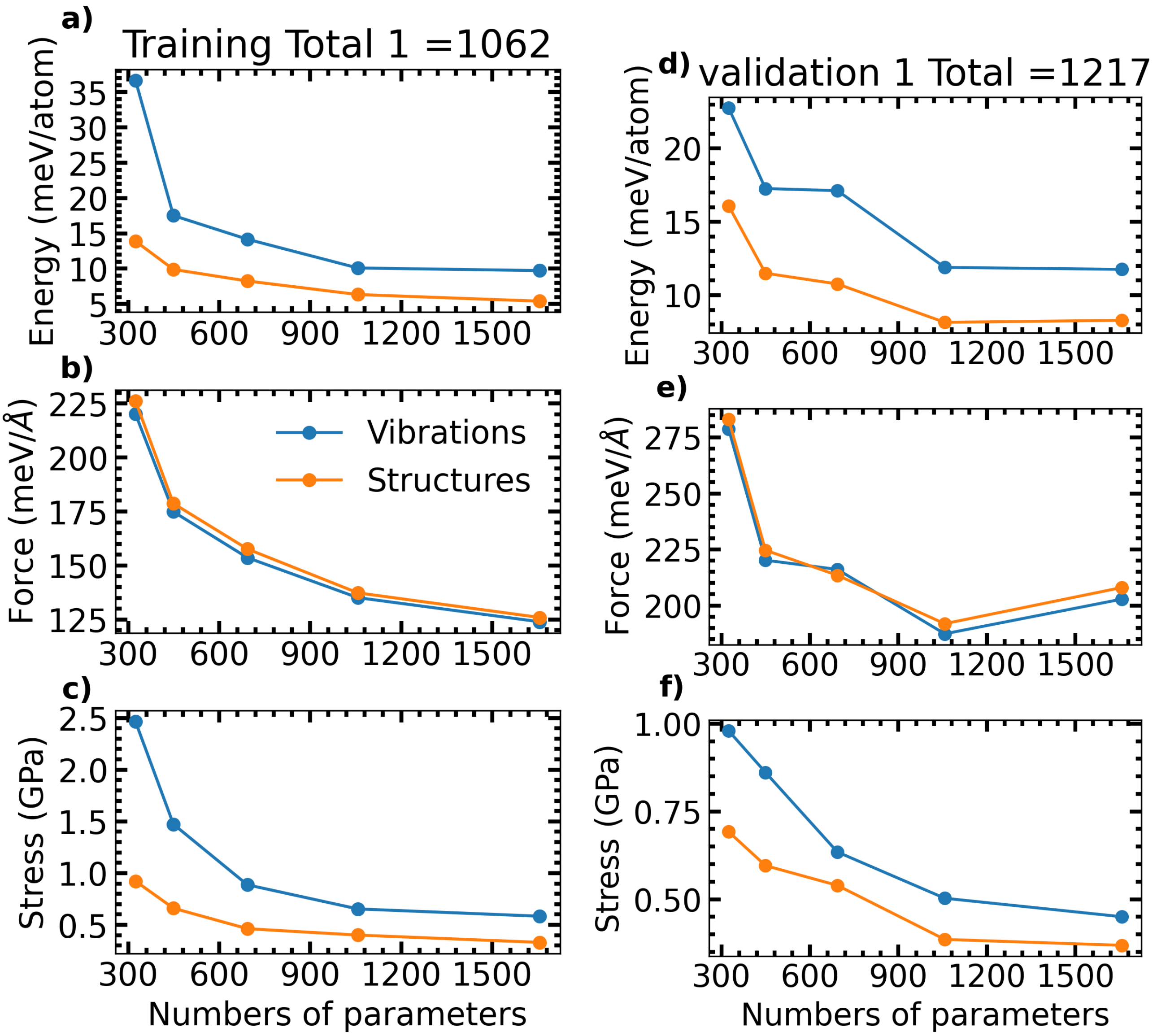

Fig. S5: Comparison of RMSE in energy, forces, and stress
We asses the RMSE errors with respect to training mode and the number of degrees of freedom. This is conducted separately on the unified training set and validation set 1. It is evident that the training mode reduces the RMSE for both energy and stress, with a noticeable decrease in RMSE as the number of parameters increases. However, the training mode has no influence on the force RMSE, which only decreases with the number of parameters. This figure can assist in selecting the appropriate training mode or deciding which mode to implement for training a diversified training set.

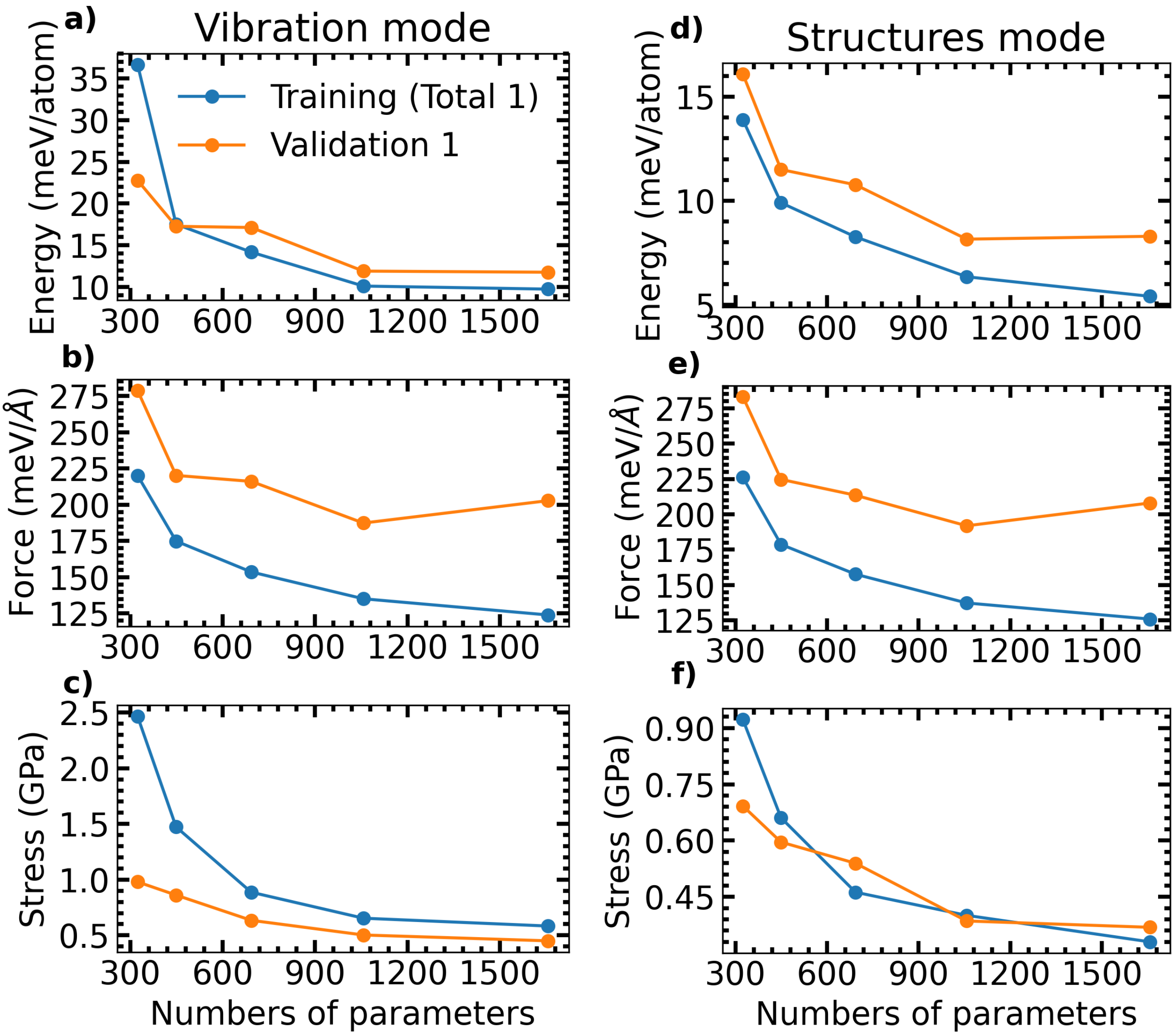

Fig. S6: Comparison of RMSE in energy, forces, and stress

In this figure, we compare the RMSE error of the training set with that of validation set 1 for each training mode while varying the number of parameters. It's important to note that validation set 1 was randomly selected concurrently with the training set from the database. As observed, neither underfitting nor overfitting is evident, even though validation set 1 is larger than the unified training set.

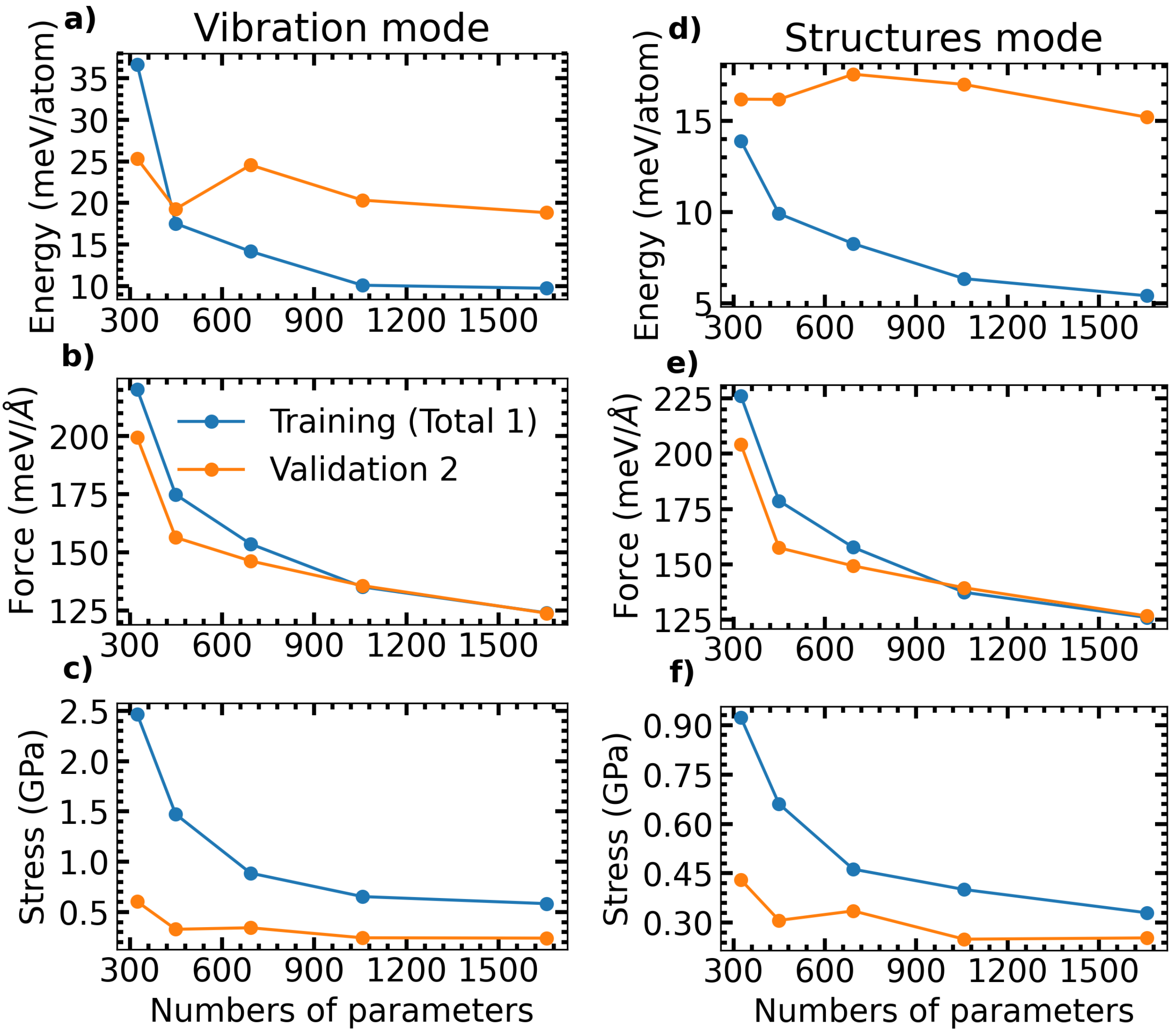

Fig. S7: Comparison of RMSE in energy, forces, and stress

A second validation set (Validation Set 2) was employed to further explore the investigation of underfitting or overfitting related issues concerning the training mode. With the exception of the energy RMSE for structure mode, all other RMSE values are indicative of a well-trained potential. Validation Set 2 was not included in the training set; it was selected from AIMD cooling/equilibration trajectories of amorphous silicon and silica. This demonstrates the transferability capability of the unified potential.

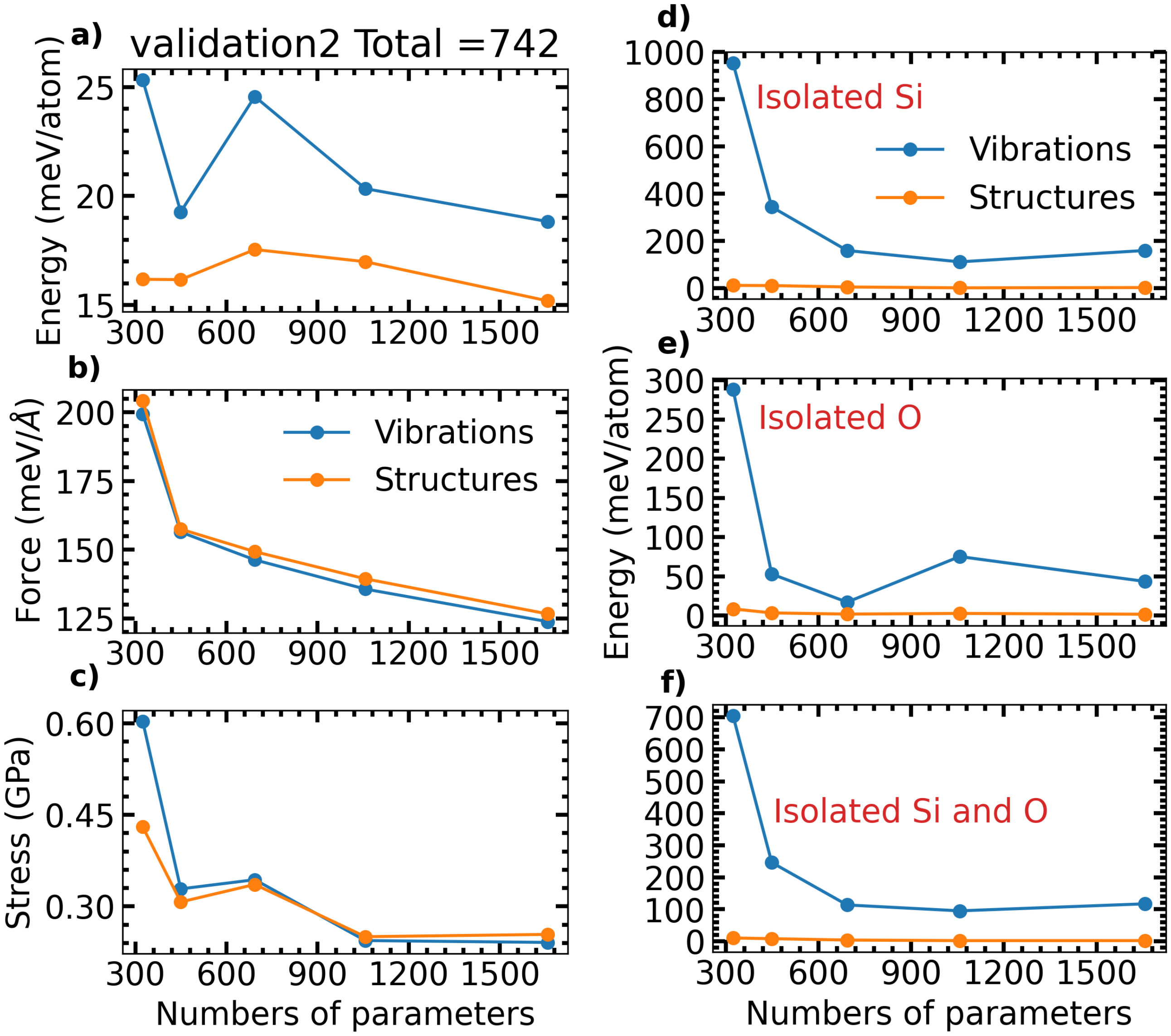

Fig. S8: Comparison of RMSE in energy, forces and stress

For the second validation set (Validation Set 2), we also assess the influence of training mode while varying the number of parameters. Overall, the structure mode outperforms the vibration mode. The graph on the right corresponds to energy errors of isolated atoms with respect to the training mode. Since we calculate cohesive energy using isolated atoms, this enables us to adopt the correct training mode. As observed, for the structures mode, RMSE never reaches zero, whereas it drops to zero when the structures mode is applied. This indicates that a normalization approach is necessary when the data is highly diversified.

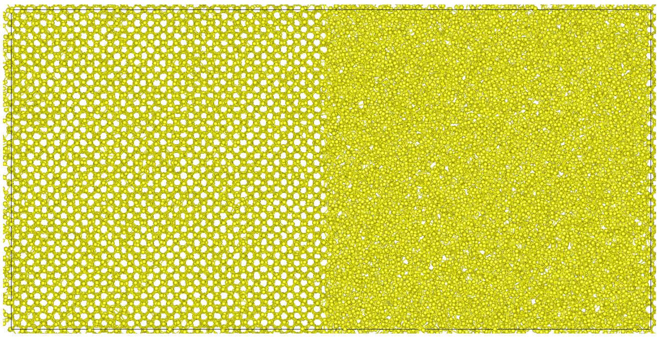

Fig. S9: Initial state of the simulation block containing both the solid and liquid phases used for the melting point simulation.

To predict the silicon crystal's melting behavior, we employed a simulation block consisting of a 12 x 12 x 12 unit cell, housing a total of 13,824 atoms. Within this framework, we considered two distinct boxes: one with solid density and another with liquid density. These boxes were meticulously constructed, each tailored to its respective temperature using specific lattice parameters. Our simulation procedure began with the independent equilibration of the solid and liquid phases. Before bringing the solid box into contact with the liquid phase, we applied a slight strain in the x and y directions. Subsequently, we conducted a comprehensive solid-liquid coexistence simulation, utilizing the NPT ensemble. The outcomes of this simulation were indicative of either solid growth or liquid expansion, contingent on the temperature (T) being either below or above the melting point. A visual representation of the simulation block used for this coexistence simulation can be found in Fig S9.

Tab. S7: First refined and optimized training set (set 1)

First refined and optimized training dataset derived from extensive uncurated database. This set was obtained through curation and optimization as described in the main text, and detailed from Tab. S3 to Fig. S6. This set was used for preliminary testing and training, as shown in Fig. S5 to Fig. S8. The distribution of the number of atoms is presented in Fig. S1 (a). 52% of the configurations presented in Tab. V of the main text, with the atom distribution shown in Fig. S1 (b), are taken from this set.

Cleaned and opitmized subset	Total
Vacancy & divacancy	51
Interstitial	34
Stacking fault	40
Others (dislocation, embedded dimer, slab SiO_2)	45
Isolated atom Si and O	2
Deformation Si	111
Deformation SiO_2	161
Disordered Si	
(85 % liquid and 15 % low temperature AIMD)	272
Liquid SiO_2	325
Oxygen molecules	21
Unified training set	1062

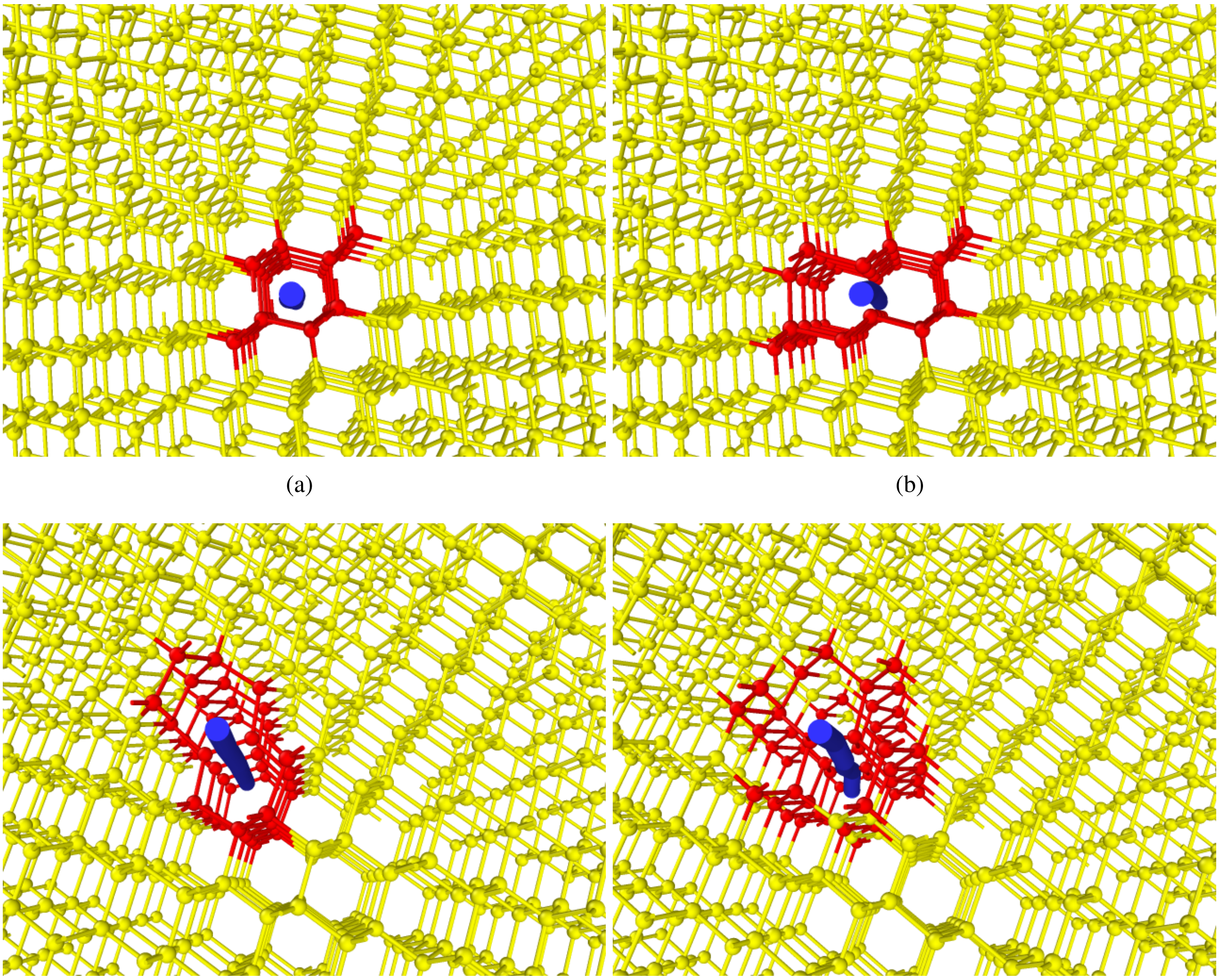

(a) (b) (c) (d)

Fig. S10: Illustrations of screw dislocation core structures obtained using the unified potential
(a) A-core, (b) B-core (which relaxes to A-core), (c) C_1 core extracted after only 5 steps of relaxation, and (d) C_2 core obtained directly from the relaxation of C_1 core. Notably, manual reconstruction, as required in previous studies such as [18] and [19], is not necessary. Atoms in close proximity to the core position are highlighted in red.The blue line represents the dislocation line.

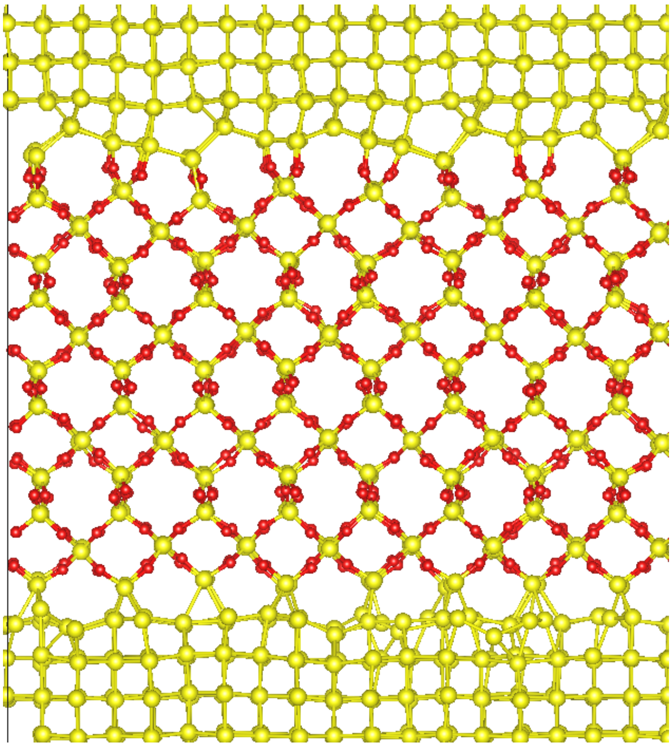

Fig. S11: Illustrations interface configurations.
A larger model of the Si/SiO_2 interface was obtained from energy and force minimization using the MTP potential

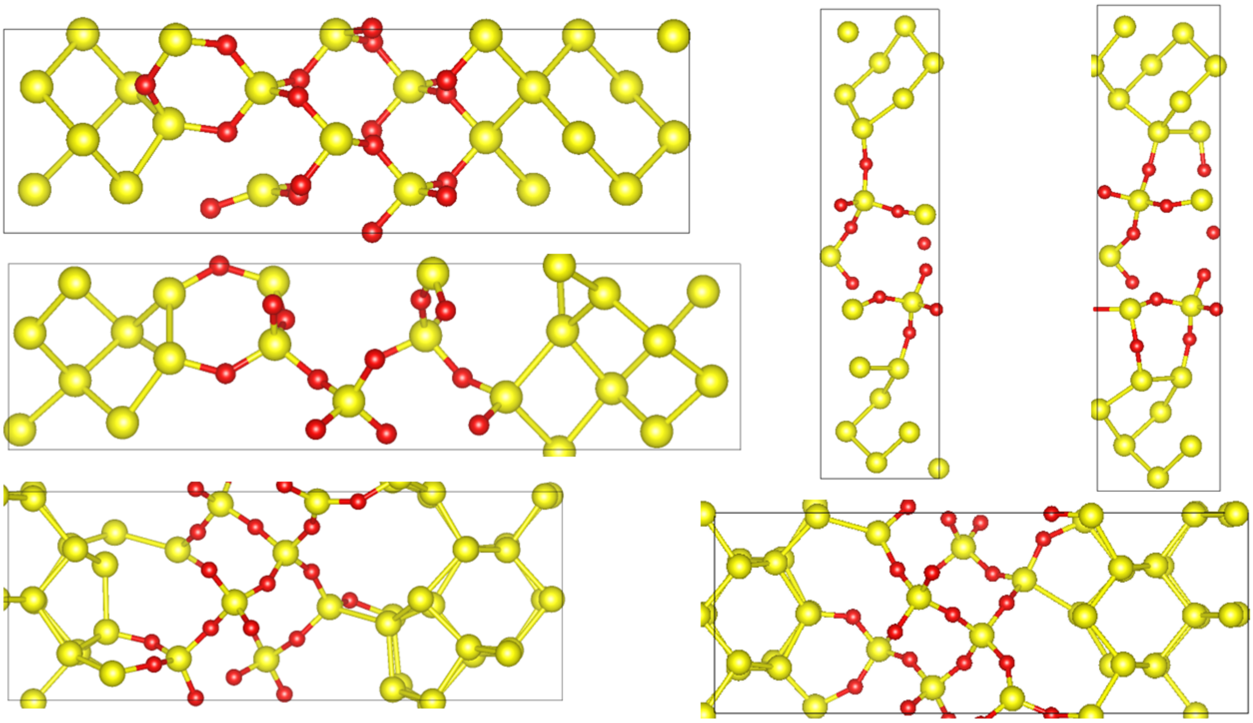

Fig. S12: Illustrations interface configurations.
The small model of Si/SiO_2 interface that was built directly from non-replicated unit cells of silicon crystal, alpha-quartz, and beta-cristobalite. These resulting configurations were obtained from energy and force minimization using the MTP potential

Tab. S8: Compositon of silica database

This table provides details on the composition of the silica database. The silicon database is also presented in [4] .

α-quartz			
Content	Replication	Atom/cell	Total
Bulk	1x1x1	9	1
Bulk deformations (tensile, compression, shear)	1x1x1	9	124
Oxygen vacancies	2x2x2	71	19
Silicon vacancies	2x2x2	71	18
Oxygen interstitial	2x2x2	72	20
Silicon interstitial	2x2x2	72	27
Dioxygen interstitial	2x2x2	73	23
Surfaces & others (0001), (-1100)	1x1x8, 2x1x8 2x2x8	8, 12, 16	2
Stacking fault	1x3x1	36	46
Random displacement	1x1x1, 2x2x2 3x3x3	9, 72,243	8
β-quartz			
Bulk	1x1x1	9	1
Bulk deformations	1x1x1	9	94
Oxygen vacancies	2x2x2	95	1
Silicon vacancies	2x2x2	95	1
Random displacement	2x2x2,3x3x3	72, 243	4
α-cristobalite			
Bulk	1x1x1	2	1
Bulk deformations	1x1x1	12	71
Oxygen vacancies	2x2x2	95	4
Silicon vacancies	2x2x2	95	4
Amorphous	2x2x2	96	3
Random displacement	1x1x1, 2x2x2 3x3x3	12, 96,324	9
β-cristobalite			
Bulk	1x1x1	6, 24	2
Bulk deformations	1x1x1	6, 24	109
Oxygen vacancies	2x2x2	192	2
Silicon vacancies	2x2x2	192	2
MD 300K	2x2x2	48	15
Liquids MD 3600K, 5000K, 8000K	2x2x2	192	16
Liquids MD ab initio 2000K, 3600K, 4600K	2x2x2	48	31
Amorphous	2x2x2	192	88
Random displacement	1x1x1, 2x2x2 3x3x3	24, 192, 648	9
α-tridymite			
Bulk	1x1x1	12	1
Bulk deformations	1x1x1	12	78
Oxygen vacancies	2x2x2	95	1
Silicon vacancies	2x2x2	95	1
Random displacement	1x1x1	12	10

**α-cristobalite II**			
Bulk	1x1x1	24	1
Bulk deformations	1x1x1	24	8
Liquids MD ab initio 4000K	1x1x1	24	9
Random displacement	1x1x1	24	2
**α-cristobalite-212121**			
Bulk	1x1x1	24	1
Bulk deformations	1x1x1	24	8
Random displacement	1x1x1	24	2
**β-cristobalite-I42d**			
Bulk	1x1x1	24	1
Bulk deformations	1x1x1	24	8
Random displacement	1x1x1	24	2
**β-cristobalite-213**			
Bulk	1x1x1	12	1
Bulk deformations	1x1x1	12	8
Random displacement	1x1x1	12	2
**Chabazite**			
Bulk	1x1x1	108	1
Bulk deformations	1x1x1	108	8
Random displacement	1x1x1	108	2
**Coesite-II**			
Bulk	1x1x1	24	1
Bulk deformations	1x1x1	24	8
Random displacement	1x1x1	24	2
**Octatetracontaoxo-tetraicosasilicate**			
Bulk	1x1x1	288	1
Bulk deformations	1x1x1	288	8
Random displacement	1x1x1	288	2
**Silica zeolite-2**			
Bulk	1x1x1	36	1
Bulk deformations	1x1x1	36	8
Random displacement	1x1x1	36	2
**Silica zeolite-GUS-1**			
Bulk	1x1x1	96	1
Bulk deformations	1x1x1	96	8
Random displacement	1x1x1	96	2

**Silica zeolite-ZSM-5**			
Bulk	1x1x1	288	1
Bulk deformations	1x1x1	288	8
Random displacement	1x1x1	288	2
**Monoclinic low tridymite**			
Bulk	1x1x1	72	1
Bulk deformations	1x1x1	72	8
Random displacement	1x1x1	72	2
**Orthorhombic high tridymite**			
Bulk	1x1x1	72	1
Bulk deformations	1x1x1	72	8
Random displacement	1x1x1	72	2
**Superstructure monoclinic tridymite**			
Bulk	1x1x1	72	1
Bulk deformations	1x1x1	72	8
Random displacement	1x1x1	72	2
**Triclinic tridymite**			
Bulk	1x1x1	72	1
Bulk deformations	1x1x1	72	8
Random displacement	1x1x1	72	2
**α-2D**			
Bulk	1x1x1	12	1
Bulk deformations	1x1x1	12	48
Oxygen vacancies	2x2x2	108	4
Silicon vacancies	2x2x2	108	3
Random displacement	2x2x2	108	2
**β-2D**			
Bulk deformations	1x1x1	12	48
Oxygen vacancies	6x6x6	108	4
Silicon vacancies	6x6x6	108	3
Random displacement	6x6x6	108	2
**δ-2D**			
Bulk	1x1x1	3	1
Bulk deformations	1x1x1	3	48
Oxygen vacancies	2x2x2	108	4
Silicon vacancies	2x2x2	108	5
Random displacement	2x2x2	108	2

	γ−2D		
Bulk	1x1x1	12	1
Bulk deformations	1x1x1	12	48
Oxygen vacancies	2x2x2	108	4
Silicon vacancies	2x2x2	108	8
Random displacement	2x2x2	108	2
	β-tridymite		
Bulk	1x1x1	12	1
Bulk deformations	1x1x1	12	81
Oxygen vacancies	2x2x2	95	1
Silicon vacancies	2x2x2	95	1
Random displacement	1x1x1	12	10
	Keatite		
Bulk	1x1x1	36	1
Bulk deformations	1x1x1	36	80
Oxygen vacancies	2x2x2	287	1
Silicon vacancies	2x2x2	287	1
Random displacement	1x1x, 2x2x2	36, 288	7
	Coesite		
Bulk	1x1x1	24	1
Bulk deformations	1x1x1	24	101
Oxygen vacancies	2x2x2	191	2
Silicon vacancies	2x2x2	191	2
Random displacement	1x1x1, 2x2x2	24, 192	7
	Moganite		
Bulk	1x1x1	18	1
Bulk deformations	1x1x1	18	101
Oxygen vacancies	2x2x2	143	2
Silicon vacancies	2x2x2	143	1
Random displacement	1x1x1, 2x2x2	18, 144	7
	Stishovite		
Bulk	1x1x1	6	1
Bulk deformations	1x1x1	6	83
Oxygen vacancies	3x3x3	161	2
Silicon vacancies	3x3x3	161	2
Random displacement	2x2x2, 3x3x3	48, 162	4
	Seifertite		
Bulk	1x1x1	12	1
Bulk deformations	1x1x1	12	83
Oxygen vacancies	2x2x2	95	2
Silicon vacancies	2x2x2	95	2
Random displacement	1x1x1, 2x2x2	12, 96	7

References

[1] Thomas J Lenosky, Babak Sadigh, Eduardo Alonso, Vasily V Bulatov, Tomas Diaz de la Rubia, Jeongnim Kim, Arthur F Voter, and Joel D Kress. Highly optimized empirical potential model of silicon. *Modelling and Simulation in Materials Science and Engineering*, 8(6):825, 2000.

[2] T Kumagai, S Izumi, S Hara, and S Sakai. Development of bond-order potentials that can reproduce the elastic constants and melting point of silicon for classical molecular dynamics simulation. *Computational materials science*, 39(2):457–464, 2007.

[3] JS Kasper and SM Richards. The crystal structures of new forms of silicon and germanium. *Acta Crystallographica*, 17(6):752–755, 1964.

[4] K. Zongo, L. K. Béland, and C. Ouellet-Plamondon. First-principles database for fitting a machine-learning silicon interatomic force field. *MRS Advances*, 7(2-3):39–47, February 2022.

[5] Anubhav Jain, Shyue Ping Ong, Geoffroy Hautier, Wei Chen, William Davidson Richards, Stephen Dacek, Shreyas Cholia, Dan Gunter, David Skinner, Gerbrand Ceder, et al. Commentary: The materials project: A materials genome approach to accelerating materials innovation. *APL materials*, 1(1), 2013.

[6] Robert T Downs and Michelle Hall-Wallace. The american mineralogist crystal structure database. *American Mineralogist*, 88(1):247–250, 2003.

[7] Stefano Curtarolo, Wahyu Setyawan, Gus LW Hart, Michal Jahnatek, Roman V Chepulskii, Richard H Taylor, Shidong Wang, Junkai Xue, Kesong Yang, Ohad Levy, et al. Aflow: An automatic framework for high-throughput materials discovery. *Computational Materials Science*, 58:218–226, 2012.

[8] Pierre Hirel. Atomsk: A tool for manipulating and converting atomic data files. *Computer Physics Communications*, 197:212–219, 2015.

[9] Koichi Momma and Fujio Izumi. Vesta: a three-dimensional visualization system for electronic and structural analysis. *Journal of Applied Crystallography*, 41(3):653–658, 2008.

[10] Alexander Stukowski. Visualization and analysis of atomistic simulation data with ovito–the open visualization tool. *Modelling and Simulation in Materials Science and Engineering*, 18(1):015012, 2009.

[11] Anton Kokalj. Xcrysden—a new program for displaying crystalline structures and electron densities. *Journal of Molecular Graphics and Modelling*, 17(3-4):176–179, 1999.

[12] Frank H Stillinger and Thomas A Weber. Computer simulation of local order in condensed phases of silicon. *Physical review B*, 31(8):5262, 1985.

[13] BWH Van Beest, Gert Jan Kramer, and RA Van Santen. Force fields for silicas and aluminophosphates based on ab initio calculations. *Physical Review Letters*, 64(16):1955, 1990.

[14] Graeme Henkelman, Blas P Uberuaga, and Hannes Jónsson. A climbing image nudged elastic band method for finding saddle points and minimum energy paths. *The Journal of Chemical Physics*, 113(22):9901–9904, 2000.

[15] Evgeny V Podryabinkin and Alexander V Shapeev. Active learning of linearly parametrized interatomic potentials. *Computational Materials Science*, 140:171–180, 2017.

[16] Ivan S Novikov, Konstantin Gubaev, Evgeny V Podryabinkin, and Alexander V Shapeev. The mlip package: moment tensor potentials with mpi and active learning. *Machine Learning: Science and Technology*, 2(2):025002, 2020.

[17] Michael Guerette and Liping Huang. A simple and convenient set-up for high-temperature brillouin light scattering. *Journal of Physics D: Applied Physics*, 45(27):275302, 2012.

[18] Xiaona Huang, Yong-Jie Hu, and Qi An. Locking of screw dislocations in silicon due to core structure transformation. *The Journal of Physical Chemistry C*, 125(44):24710–24718, 2021.

[19] Julien Guénolé, Julien Godet, and Laurent Pizzagalli. Determination of activation parameters for the core transformation of the screw dislocation in silicon. *Modelling and Simulation in Materials Science and Engineering*, 18(6):065001, 2010.
